# Using Virtual Reality Head-Mounted Displays to Assess Skills in Emergency Medicine: Validity Study

**DOI:** 10.2196/45210

**Published:** 2023-06-06

**Authors:** Marie Høxbro Knudsen, Niklas Breindahl, Tor-Salve Dalsgaard, Dan Isbye, Anne Grethe Mølbak, Gerhard Tiwald, Morten Bo Søndergaard Svendsen, Lars Konge, Joanna Bergström, Tobias Todsen

**Affiliations:** 1 Department of Otorhinolaryngology, Head and Neck Surgery and Audiology Rigshospitalet University of Copenhagen Copenhagen Denmark; 2 Prehospital Center Region Zealand Næstved Denmark; 3 Copenhagen Academy for Medical Education and Simulation Center for HR and Education Copenhagen Denmark; 4 Department of Computer Science University of Copenhagen Copenhagen Denmark; 5 Department of Anesthesia, Section 6011 Centre of Head and Orthopeadics Rigshospitalet, University of Copenhagen Copenhagen Denmark; 6 Department of Clinical Medicine University of Copenhagen Copenhagen Denmark; 7 Emergency Department Zealand University Hospital Køge Denmark

**Keywords:** virtual reality, simulation-based education, undergraduate medical education, emergency medicine, assessment, acute medicine, Messick framework, medical education, head-mounted display, medical student, emergency

## Abstract

**Background:**

Many junior doctors must prepare to manage acutely ill patients in the emergency department. The setting is often stressful, and urgent treatment decisions are needed. Overlooking symptoms and making wrong choices may lead to substantial patient morbidity or death, and it is essential to ensure that junior doctors are competent. Virtual reality (VR) software can provide standardized and unbiased assessment, but solid validity evidence is necessary before implementation.

**Objective:**

This study aimed to gather validity evidence for using 360-degree VR videos with integrated multiple-choice questions (MCQs) to assess emergency medicine skills.

**Methods:**

Five full-scale emergency medicine scenarios were recorded with a 360-degree video camera, and MCQs were integrated into the scenarios to be played in a head-mounted display. We invited 3 groups of medical students with different experience levels to participate: first- to third-year medical students (novice group), last-year medical students without emergency medicine training (intermediate group), and last-year medical students with completed emergency medicine training (experienced group). Each participant’s total test score was calculated based on the number of correct MCQ answers (maximum score of 28), and the groups’ mean scores were compared. The participants rated their experienced presence in emergency scenarios using the Igroup Presence Questionnaire (IPQ) and their cognitive workload with the National Aeronautics and Space Administration Task Load Index (NASA-TLX).

**Results:**

We included 61 medical students from December 2020 to December 2021. The experienced group had significantly higher mean scores than the intermediate group (23 vs 20; *P*=.04), and the intermediate group had significantly higher scores than the novice group (20 vs 14; *P*<.001). The contrasting groups’ standard-setting method established a pass-or-fail score of 19 points (68% of the maximum possible score of 28). Interscenario reliability was high, with a Cronbach α of 0.82. The participants experienced the VR scenarios with a high degree of presence with an IPQ score of 5.83 (on a scale from 1-7), and the task was shown to be mentally demanding with a NASA-TLX score of 13.30 (on a scale from 1-21).

**Conclusions:**

This study provides validity evidence to support using 360-degree VR scenarios to assess emergency medicine skills. The students evaluated the VR experience as mentally demanding with a high degree of presence, suggesting that VR is a promising new technology for emergency medicine skills assessment.

## Introduction

Many junior doctors are not sufficiently prepared to handle critically ill patients in the emergency department [1-4]. The environment is stressful, and rapid diagnostic workup and treatment of acutely ill patients are needed for successful outcomes [5-7]. During the undergraduate clinical rotations, the supervision and assessment of clinical performance in a workplace setting for high-risk, low-frequency emergencies are limited [8]. Junior doctors must be competent to ensure patient safety, as overlooking symptoms and making wrong choices may lead to substantial patient morbidity or death. Simulation-based education is often used for training and skills assessment but is resource demanding in cost and faculty time. Virtual reality (VR) videos on a head-mounted display (HMD) can provide a low-cost, time-efficient supplement for full-scale simulation and induce the stressful experience of being present in an emergency department setting [9-11].

The HMD allows a first-person view of the emergency room and interaction with the patient and other team members. A 360-degree VR video is recorded in all directions, giving the user a complete 360-degree view. The authentic experience of being present is further induced by recording in an actual emergency department [9-11]. VR software can provide standardized and unbiased assessment, but validity evidence must be explored before implementation. We developed a VR application (360MedQuest) where multiple-choice questions (MCQs) were integrated into a 360-degree video to present realistic full-scale emergency medicine cases in VR.

The primary aim of this study was to explore the validity of evidence according to the 5 sources in Messick’s [12] validity framework. The secondary aim was to explore user acceptability by measuring the student’s presence experience and workload during the 360-degree VR scenarios.

## Methods

### Ethical Considerations

We conducted an experimental study at Copenhagen Academy for Medical Education and Simulation (CAMES), Copenhagen, Denmark, from December 2020 to December 2021. The Committee on Health Research Ethics in the Capital Region of Denmark waived the need for ethical approval (journal number H-20037984). Data management and processing were approved (Pactius ID number P-2022-63).

### Development of the VR Application

Five emergency scenarios and 1 introduction scenario were recorded in high-resolution (10K) stereoscopic 360-degree (360VR) video with a Titan 8-lens VR camera (Insta360). The 6 videos were stitched to single 360-degree stereoscopic VR video clips edited in Final Cut Pro (Apple, Inc) video-editing software to generate one long VR video for each scenario. The 360MedQuest VR app was developed in Unity (Unity Technologies) to integrate 360-degree videos in an interactive VR environment with integrated MCQs presented during the 360VR video scenarios. Oculus Quest 1 and 2 (Meta Platforms, Inc) were used as VR hardware to allow high-resolution and immersion interaction with the videos by the students.

Video recordings of the different emergency scenarios occurred in a full-scale simulation room at CAMES, Herlev, Denmark. Teachers and students from the simulation center played the roles of patients, patient relatives, and health care staff in the emergency department. The videos were recorded from the view of an attending emergency physician supervising a junior doctor who performed the examination and clinical procedures. The viewer was addressed directly in the 360VR video and talked about in second person to increase the immersion.

**Figure 1:**
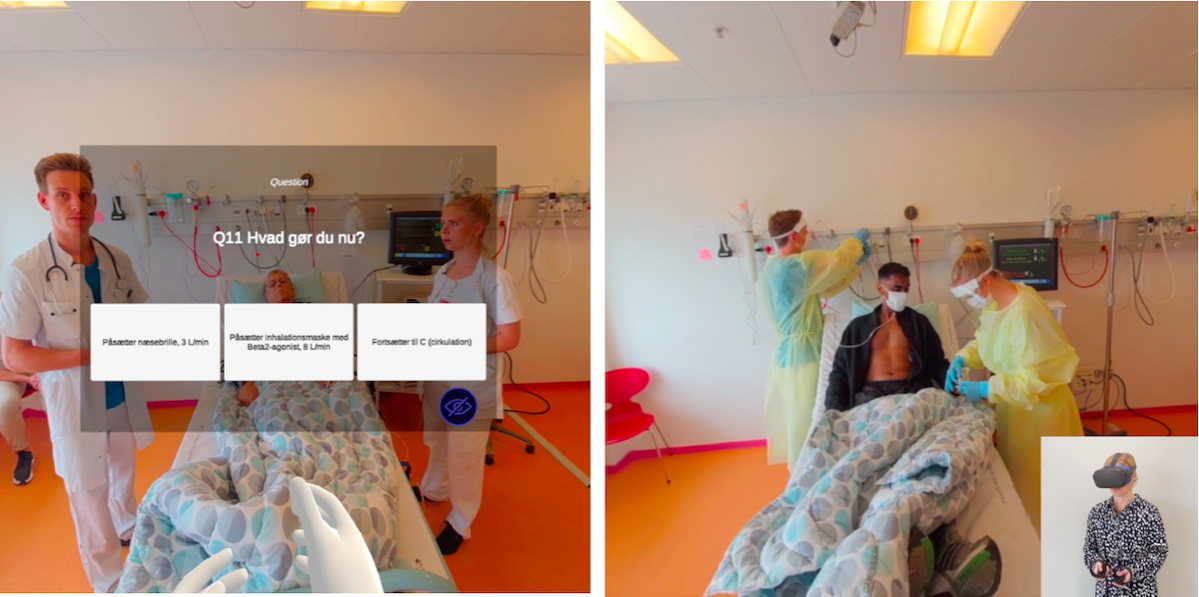
Snapshots from the anaphylaxis and COVID-19 scenarios. The patient is in the middle of the picture, the junior doctor on the left, and the nurse on the right. Left: Screenshot from the anaphylaxis scenario. The patient's next of kin sits behind the junior doctor. The controllers are illustrated in the VR environment as 2 hands. An MCQ pauses the scenario: “Q11 What do you do now? Use an oxygen catheter in the nose, 3 L/min; Put on an inhalation mask with Beta2 agonist, 6 L/min; Continue to C (circulation)." Right: Screenshot from the COVID-19 scenario. A participant wearing a VR head-mounted display is shown as a picture in a picture with the experienced scenario. MCQ: multiple-choice question; VR: virtual reality.

The scenarios consisted of one long video sequence paused by an MCQ overlaid onto the video image in VR (Figure 1) when the participant needed to make decisions on diagnostic and treatment interventions. Each MCQ had 3 options with a single correct answer. The participants chose their answers with controllers shown as 2 hands in the HMD. Following each MCQ, the correct answer was revealed as a narrator’s voice supervising the junior doctor, and the scenario continued independent of the chosen answer. A correct answer resulted in 1 point; an incorrect answer resulted in 0 points. A total score was then calculated based on the sum of correct MCQ answers from the 5 scenarios (range 0-28 points). Participants were excluded from analyses if they did not complete all 5 scenarios. See Multimedia Appendix 1 for a scenario demonstration.

### Participants

Medical students from the University of Copenhagen, Denmark, with different experience levels were invited to participate: (1) first- to third-year medical students with no clinical experience (novice group), (2) sixth-year medical students without emergency medicine training (intermediate group), and (3) sixth-year medical students after completing a 5-week intensive course in emergency medicine including 1 day with full-scale simulation-based training (experienced group). All participants volunteered and gave verbal and written informed consent before enrollment.

### Validation of VR Assessments

Using Messick’s [12] validity framework, we explored the validity evidence for using 360VR scenarios with integrated MCQs to assess emergency medicine skills. Messick’s framework consists of 5 sources of validity evidence regarding content, response process, internal structure, relations to other variables, and consequences [12,13].

#### Content

Five different emergency medicine scenarios were developed: (1) COVID-19 respiratory distress syndrome, (2) trauma and hypovolemic shock, (3) myocardial infarction with in-hospital cardiac arrest, (4) anaphylaxis, and (5) hypoglycemia in the unconscious patient. Besides the 5 emergency scenarios, a simple introduction scenario with 2 questions was created. The scenarios and MCQs were written by a multidisciplinary expert group (a consultant in acute medicine, a consultant in anesthesiology, a trauma surgery consultant, and a professor of medical education) and peer-student instructors in emergency medicine at CAMES. The content of scenarios and MCQs were based on the curriculum for the course in emergency medicine at the University of Copenhagen and relevant guidelines from the European Resuscitation Council and the Danish Society of Anesthesiology and Intensive Care Medicine.

#### Response Process

The participants received standardized VR equipment instructions, including a short introduction scenario with simple nonmedical questions. Afterward, the participants played the scenarios in dedicated rooms. Each participant went through the same scenarios in the same order and only once. If needed, participants were allowed to ask for technical help only during the VR scenarios from an on-site instructor. No assistance was offered regarding the correct answers.

#### Internal Structure

We calculated the participants’ number of correct MCQ answers for each scenario, and Cronbach α was calculated to assess the interscenario reliability. A Cronbach α>0.80 indicates a high reliability that is adequate for summative assessment [14].

#### Relations to Other Variables

A one-way ANOVA was used to compare the mean test score between the 3 groups. Independent sample 2-tailed *t* tests were used to make direct comparisons between the novice and intermediate groups and between the intermediate and experienced groups. *P*<.05 was considered statistically significant. All statistical analyses were performed using a software package (PASW, version 26.0; SPSS Inc), and 2-sided significance levels of .05 were used for all analyses.

#### Consequences

A pass-or-fail score was established using the contrasting groups’ standard-setting method based on the test scores from the novice and experienced groups. Two bell-shaped curves were plotted based on the means and SDs of the 2 groups. The pass-or-fail score is defined as the intersection between the 2 groups. The standard-setting method is described in the paper from Jørgensen et al [15], including a Microsoft Excel file to plot the curves and establish the score.

The consequences of this threshold were explored by reporting the number of false positives (ie, participants from the novice group who passed the test) and false negatives (ie, participants from the experienced group who failed the test).

### VR Perception

After completing the scenarios, the participants rated their perceived presence and cognitive workload in the emergency scenarios. Presence is defined as a person’s subjective experience of being physically present in a digital environment [16,17]. The Igroup Presence Questionnaire (IPQ) [18] measured the sense of presence in the virtual surroundings. It is a validated questionnaire consisting of 14 questions about general presence, spatial presence (feeling present in a mediate world), involvement, and experienced realism that is answered on a 7-point Likert scale [19].

IPQ was originally constructed in German [18]; the English version was used in this study as our participants were medical students who were used to English from textbooks and lectures at the university. The workload in VR was measured with the National Aeronautics and Space Administration Task Load Index (NASA-TLX) [20], assessing experienced performance, mental demand, experienced effort, physical demand, temporal demand, and frustration during the scenarios [21,22].

The Kruskal-Wallis test was used to compare data from the IPQ and NASA-TLX between the 3 groups. The Wilcoxon rank-sum test was used for direct comparison between each group.

*P*<.05 was considered statistically significant. All statistical analyses were conducted in R (version 4.2.3; R Foundation for Statistical Computing), and 2-sided significance levels of .05 were used for all analyses.

## Results

### User Statistics

**Figure 2:**
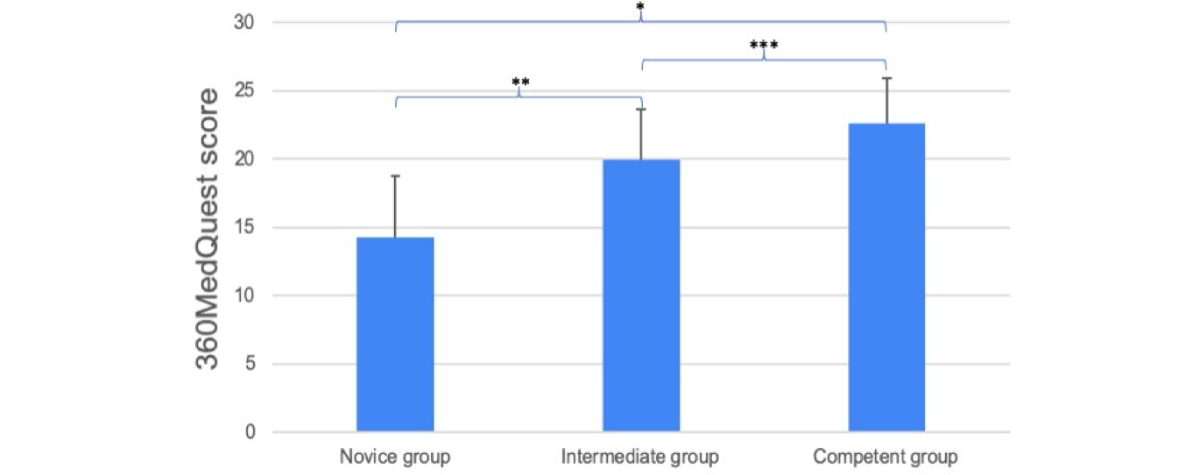
360MedQuest score (mean total test score) for the 3 groups, including mean and SD. *P<.001, **P<.001, and ***P=.04.

We enrolled 61 medical students: 21 (34%) in the novice group, 20 (33%) in the intermediate group, and 20 (33%) in the experienced group. Two (3%) participants (1 from the intermediate group and 1 from the experienced group) interrupted their scenarios because they did not have time to complete all 5 scenarios and were excluded. Baseline characteristics for the 59 included participants are presented in Table 1. The mean total test scores (360MedQuest score) were 14.2 (SD 4.5), 19.9 (SD: 3.7), and 22.7 (SD: 3.4) for the novice, intermediate, and experienced groups, respectively, all normally distributed. ANOVA demonstrated a significant difference between all 3 groups (*P*<.001). The score of the experienced group was significantly higher than that of the intermediate group (*P*=.04), and the intermediate group scored significantly higher than the novice group (*P*<.001; Table 1 and Figure 2).

**Figure 3:**
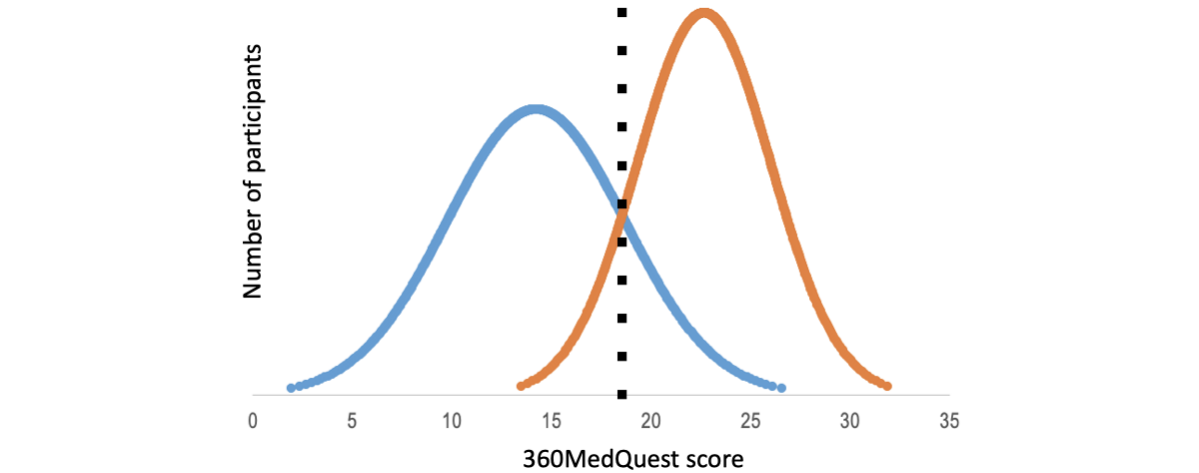
A pass-or-fail score of 19 points (68% of the maximum possible score of 28) was established by contrasting groups’ method. Blue curve: novice group; orange curve: experienced group. 360MedQuest score: mean total test score.

The internal consistency reliability across all items was high, with a Cronbach α of 0.82. The contrasting groups’ method established a pass-or-fail score of 19 points (68% of the maximum possible score of 28; Figure 3). As a consequence of the standard setting, 4 (19%) out of 21 participants in the novice group passed the test, and 1 (5%) out of 19 participants in the experienced group failed.

**Table 1:** Baseline data, mean total score, SD, and pass-or-fail score for the 3 groups.

	Novice group (n=21)	Intermediate group (n=19)	Experienced group (n=19)
Age (years), mean (SD)	24.4 (4.07)	27.4 (2.17)	27.0 (1.89)
Sex (female), n (%)	12 (57)	11 (58)	15 (79)
No experience with VR^a^, n (%)	11 (52)	12 (63)	11 (58)
Total MCQ^b^ score, mean (SD)	14.24 (4.48)	19.95 (3.66)	22.58 (3.35)
Number of participants passing, n (%)	4 (19%)	14 (74%)	18 (95%)

^a^VR: virtual reality.

^b^MCQ: multiple-choice question.

### VR Perception

Data from the IPQ and NASA-TLX were not normally distributed and were calculated with nonparametric tests: Kruskal-Wallis and Wilcoxon rank-sum tests.

#### IPQ Data

The participants rated the degree of presence in the VR simulation with a mean general presence of 6.0 (on a scale from 1-7) without significant difference between the novice, intermediate, and experienced groups (*P*=.64). The intermediate and experienced groups rated experienced realism as 4.3 and 4.4, respectively (on a scale from 1-7). The groups had no significant differences regarding the spatial presence (*P*=.11) and degree of involvement (*P*=.22; Table 2).

**Table 2:** Data for each questionnaire: Igroup Presence Questionnaire (IPQ) and National Aeronautics and Space Administration Task Load Index (NASA-TLX).

			Novice group, mean (SD)		Intermediate group, mean (SD)		Experienced group, mean (SD)		All groups, mean (SD)		Scale		*P* value^a^
**IPQ**													
	General presence	5.95 (0.78)		5.89 (0.74)		6.15 (0.80)		6.00 (0.14)		1-7		.64	
	Realness	4.59 (0.74)		4.33 (0.80)		4.38 (0.74)		4.44 (0.14)		1-7		.62	
	Spatial presence	5.51 (0.77)		5.89 (0.74)		5.63 (0.69)		5.68 (0.19)		1-7		.11	
	Involvement	4.77 (0.80)		4.59 (1.05)		5.13 (1.50)		4.83 (0.27)		1-7		.22	
**NASA-TLX**													
	How mentally demanding was the task?	12.26 (4.03)		14.00 (3.65)		13.65 (3.47)		13.30 (0.92)		1 (very low) to 21 (very high)		.36	
	How physically demanding was the task?	4.21 (3.98)		5.00 (4.74)		4.95 (4.83)		4.72 (0.44)		1 (very low) to 21 (very high)		.90	
	How hurried or rushed was the pace of the task?	7.74 (3.83)		9.94 (3.17)		8.50 (2.91)		8.73 (1.12)		1 (very low) to 21 (very high)		.21	
	How successful were you in accomplishing what you were asked to do?	12.05 (4.67)		8.78 (3.02)		7.20 (2.95)		9.34 (2.48)		1 (perfect) to 21 (failure)		.004^b^	
	How hard did you have to work to accomplish your level of performance?	13.05 (3.70)		13.11 (3.56)		11.40 (4.59)		12.52 (0.97)		1 (very low) to 21 (very high)		.52	
	How insecure, discouraged, irritated, stressed, and annoyed were you?	6.63 (4.68)		7.83 (4.88)		9.00 (5.01)		7.82 (1.18)		1 (very low) to 21 (very high)		.33	

^a^*P* values between groups were calculated using Kruskal-Wallis tests due to nonnormality.

^b^Significant *P* value <.05.

#### NASA-TLX Data

**Figure 4:**
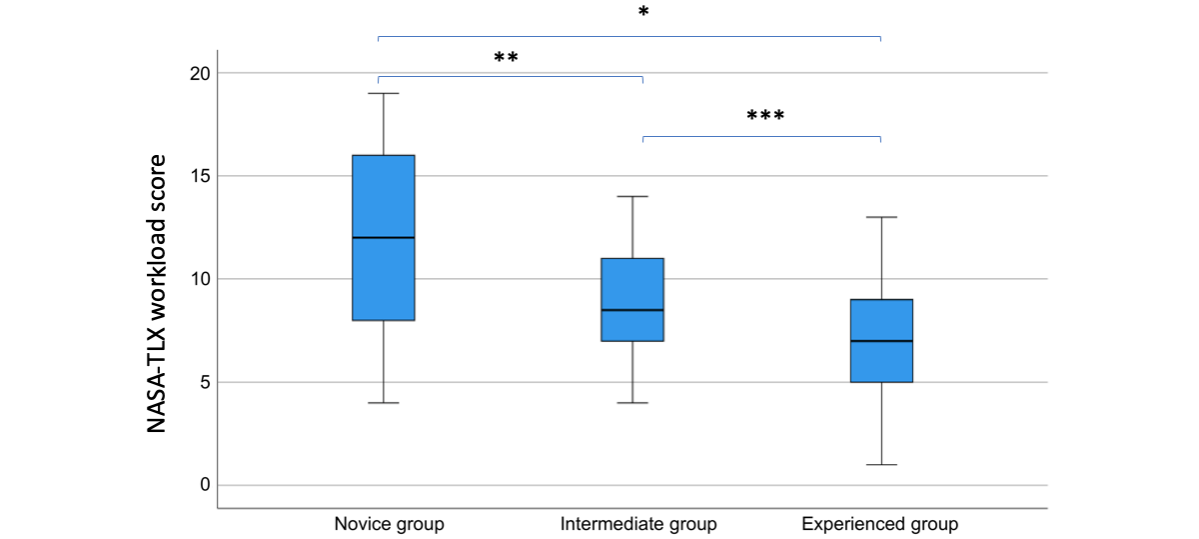
The groups’ experienced performance according to the NASA-TLX workload questionnaire. The groups with greater scores experienced more failure in their performance. Multiple pairwise comparisons between groups were conducted with the Wilcoxon rank-sum test with continuity correction. *P<.001, **P=.02, and ***P=.11. The experienced group rated their performance significantly higher than the intermediate and novice groups. NASA-TLX: National Aeronautics and Space Administration Task Load Index.

The experienced group evaluated their performance significantly higher than the novice group (*P*=.007; Figure 4). The average mental demand across groups was a NASA-TLX score of 13.3 (on a scale from 1-21), and the average experienced effort was rated as 12.5 (on a scale from 1-21). There were no significant differences between groups when evaluating physical demand (*P*=.90), temporal demand (*P*=.21), and frustration (*P*=.33) during the scenarios (Table 2).

## Discussion

### Principal Findings

This study provides validity evidence supporting the use of 360-degree VR scenarios with MCQs for assessing skills in emergency medicine among medical students investigated with Messick’s [12] validity framework. The 360MedQuest score significantly discriminated between different groups with high interscenario reliability. Further, all groups experienced a high degree of presence during the VR scenarios and experienced the tasks as mentally demanding.

A strength of our study is the experimental design, where the skills of the medical students were assessed in a standardized and reproducible way with the automatic generation of test scores eliminating the risk of human typing error [23]. Automating and standardizing the scoring system also means avoiding rater bias and that the student receives a fair score [24,25]. Furthermore, this study was investigated with the 5 steps of Messick’s [12] validity framework. The internal consistency reliability across all items was high, and a pass-or-fail score was calculated.

Another strength of this study is the high level of presence, workload, and realism experienced in the scenarios by the participants. The experience of presence and the cognitive workload was measured in the VR scenarios using validated tools: the IPQ and NASA-TLX. Presence is a quality measure to provide such a perception or experience in VR applications. Higher presence experience has been demonstrated to increase the transfer of learning from training to clinical performance [22,26]. A mean IPQ score of 6 by the participants in our study would correspond to an “Excellent Presence” compared to other studies exploring VR experiences with the IPQ score [27]. Using a 360-degree high-resolution camera, using live actors, and talking to the participant in second person may have contributed to a high degree of presence.

The intermediate and experienced groups have experience from clinical rotations and rated the simulations as realistic: average ratings of 4.3 and 4.4 (on a scale from 1-7), respectively.

The NASA-TLX measured the mental demand and the participant’s perception of their performance. The scenarios were experienced as highly demanding, with a mean score across all 3 groups of 13.3. Hertzum [21,22] conducted a meta-analysis of the NASA-TLX and made a reference scale of the degree of presence. Our mean score of 13.3 equals 66.5 on Hertzum’s [21,22] scale, which is higher than the mean score within other emergency response (ambulance services, police, and firefighting) and health care (in-hospital training of nurses, pharmacists, and physicians) services [21]. We believe 360MedQuest can induce the mental demands of work in real emergencies. On average, the experienced group achieved a significantly higher MCQ score. They rated their performance significantly higher than the novice group, meaning the participants generally understood their performance in the scenarios well (Figure 4).

### Limitations

A limitation of our study is that we only included medical students and no medical specialists for the different competency groups. However, as the MCQs were designed to assess the emergency medicine skills expected of senior medical students, we prioritized conducting a validation study including the target group. It is a strength that the test score could significantly differentiate between the 3 competence levels among medical students instead of only between novice and expert performance levels. Another limitation is that the MCQs were limited to 1 correct answer out of 3 options. Our design is less transferable to “real life” than a full-scale simulation where students can freely make treatment decisions without being limited by predecided options. According to Bloom’s Taxonomy [28], if we had created 360VR scenarios in which the student could make unrestricted decisions, it would have achieved a higher level of complexity. However, creating such an available VR environment would also significantly increase the complexity of VR software and the development costs. Instead, we suggest a more straightforward and low-cost solution that can be used for a much more realistic assessment than traditional text-based MCQ tests. We combined it with real 360-degree video scenarios to increase the transfer to a natural clinical setting. Another limitation is the risk of type I error due to multiple significance tests being performed. Although a post hoc correction method (such as the Bonferroni method) could be applied to account for the multiple tests, it would also be a highly conservative method and risk missing fundamental differences between the groups. Since we only compared scores from 3 groups, we estimated that a post hoc test was unnecessary [29].

### Comparison With Prior Work

VR is a well-established tool of training in surgery [30-32], anatomy [33,34], and other medical fields [35-37]. Still, it is mainly built on graphic animations, limiting realism and presence compared to a natural clinical setting. Previous studies have focused on a single subject and compared 360-degree videos to no intervention or traditional videos [38-40]. This study is the first to investigate the validity of the evidence of real 360-degree VR videos with integrated MCQs as a tool to assess skills in medicine. From experimental sports science, 360-degree VR videos have also been found to be a valid tool for assessing decision-making in Australian football [41].

We demonstrated that 360-degree videos with integrated MCQs can provide a high degree of immersion and presence among medical students and can be used for standardized skills assessment in a failure-safe environment [10]. Once developed, it will be inexpensive compared to full-scale simulation training, and it can be used under social distancing during a pandemic [42]. Future studies should explore if 360-degree VR scenarios with MCQs can also be used in postgraduate skills assessment and as an effective education tool.

### Conclusion

In conclusion, 360-degree VR scenarios with integrated MCQs can be used to assess emergency medicine skills among medical students. The VR experience was evaluated as mentally demanding with a high degree of presence, suggesting that VR is a promising new technology for emergency medicine skills assessment.

## Appendix

Multimedia Appendix 1 Demonstration of a scenario.

## Abbreviations

CAMES: Copenhagen Academy for Medical Education and Simulation
HMD: head-mounted display
IPQ: Igroup Presence Questionnaire
MCQ: multiple-choice question
NASA-TLX: National Aeronautics and Space Administration Task Load Index
VR: virtual reality
